# Supplementary material for: Somatic clones heterozygous for recessive disease alleles of BMPR1A exhibit unexpected phenotypes in Drosophila
Source: eLife. 2018 May 10;7:e35258. doi: 10.7554/eLife.35258 (PMC5963922; doi:10.7554/eLife.35258)
Supplement: Figure 2—source data 1. — Wild-type clones (green) showed higher BMP/DPP activity (grey) compared to their tkvC97R homozygous neighbors (red) and frequently exhibited an outgrowth phenotype. [file elife-35258-fig2-data1.docx]

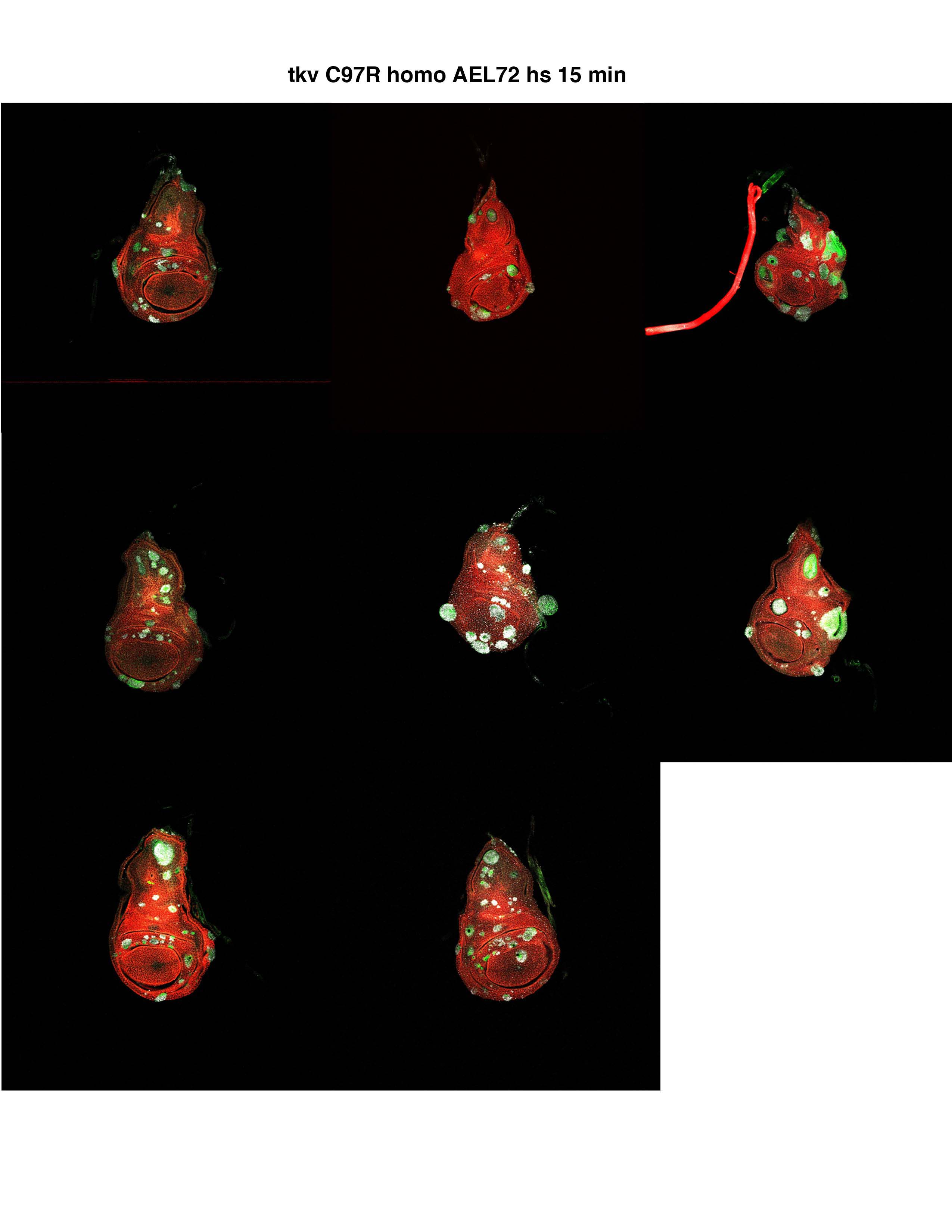


**Figure 2-source_data_1. BMP/DPP activation in *wild-type* clones within the *tkv^C97R^* homozygous background**

*Wild-type* clones (*green*) showed higher BMP/DPP activity (*grey*) compared to their *tkv^C97R^* homozygous neighbors (*red*) and frequently exhibited an outgrowth phenotype.
