# Supplementary material for: Somatic clones heterozygous for recessive disease alleles of BMPR1A exhibit unexpected phenotypes in Drosophila
Source: eLife. 2018 May 10;7:e35258. doi: 10.7554/eLife.35258 (PMC5963922; doi:10.7554/eLife.35258)
Supplement: Supplementary file 1. — EcoRI and AscI sites are underlined in 17 and 22, respectively. Lowercase characters indicate point mutations. [file elife-35258-supp1.docx]

| **Primer** | **Primer sequence (5’ to 3’)** | **Purpose** |
| --- | --- | --- |
| 1 | CTTCGGATGATTGGTTTAAAGGTG | *tkv* sgRNA construct 1 |
| 2 | AAACCACCTTTAAACCAATCATCC | *tkv* sgRNA construct 1 |
| 3 | CTTCGGAGAGCTTGTCGTCGAGGG | *tkv* sgRNA construct 2 |
| 4 | AAACCCCTCGACGACAAGCTCTCC | *tkv* sgRNA construct 2 |
| 5 | CCGGGTACCGAGCTCGAA | *tkv allele switch* *founder* donor *pHSG298* |
| 6 | GGATCCTCTAGAGT CGACCTG | *tkv allele switch* *founder* donor *pHSG298* |
| 7 | AGGTCGACTCTAGAGGATCCGCGAACGCCAATAAACA ATAATG | *tkv allele switch* *founder* donor left arm homology |
| 8 | TAGGAACTTCCTTTAAACCAATCATCCACAATC | *tkv allele switch* *founder* donor left arm homology |
| 9 | TGGTTTAAAGGAAGTTCCTATTCCGAAGTTCCTATTCT CTAGAAAGTATAGGAACTTCGTGGGGATAAGACCAACCCACTCGTCAAC | *tkv allele switch* *founder* donor *FRT* 5’-*tkv* exon |
| 10 | TGGCGGCGGCCTCGACAATCTTAATGGGCACATC | *tkv allele switch* *founder* donor *FRT* 5’-*tkv* exon |
| 11 | TAAGATTGTCGAGGCCGCCGCCAAAGAAGCCGCAGCAAAAGAAGCAGCTGCCAAGGAAGCCGCAGCGAAGATGAGTAAAGGAGAAGAACTTTTCACTGGAG | *tkv allele switch* *founder* donor rigid linker-*GFP*-*tkv 3’UTR* |
| 12 | CTACTCCGAATTCGGTTTAATTATAATTTTTTATTATTTTTAAATAAATTAACTAAC | *tkv allele switch* *founder* donor rigid linker-*GFP*-*tkv 3’UTR* |
| 13 | TATAATTAAACCGAATTCGGAGTAGTGCCCCAACTGGGGTAACCTTTGAGTTCTCTCAGTTGGGGGCGTAGGATAACTTCGTATAGCATACATTATACGAAGTTATTCGCCAAGCTTGGGCTGCATC | *tkv allele switch* *founder* donor attP-*loxP* 5’-*ubi-mCherry* |
| 14 | TATACGAAGTTATTTAACTTACATACATACTAGAATTGATC | *tkv allele switch* *founder* donor attP-*loxP* 5’-*ubi-mCherry* |
| 15 | GTATGTAAGTTAAATAACTTCGTATAGCATACATTATACGAAGTTATGCTCGCAGACCACAAGAACTAAAGTTTTGTATTAAAGC | *tkv allele switch* *founder* donor *loxP* 3’-right arm homology |
| 16 | AATTCGAGCTCGGTACCCGGCCCAGCCAATACCCAAC CAATAACCCAACC | *tkv allele switch* *founder* donor *loxP* 3’-right arm homology |
| 17 | GGCGAATTCGAAGTTCCTATTCCGAAGTTCCTATT CTCT AGAAAGTATAGGAACTTCGTG | *tkv allele switch* *cassette*  EcoRI-*FRT* 3’-*tkv* exon-rigid linker |
| 18 | GCTCACCATGGTCATCTTCGCTGCGGCTTCCTTGGCAGCTG | *tkv allele switch* *cassette*  EcoRI-*FRT* 3’-*tkv* exon-rigid linker |
| 19 | GAAGCCGCAGCGAAGATGACCATGGTGAGCAAGGGCGAGGAG | *tkv allele switch* *cassette*  mCherry |
| 20 | ACTACAAACAGCTGCTTACTTGTACAGCTCGTCCATGC CGC | *tkv allele switch* *cassette*  mCherry |
| 21 | GAGCTGTACAAGTAAGCAGCTGTTTGTAGTCTCGTTTT AGGTTTAACC | *tkv allele switch* *cassette*  *tkv* 3’UTR-*loxP*-AscI |
| 22 | GGCGGCGCGCCATAACTTCGTATAATGTATGCTATACGAAGTTATGGTACCGGTTTAATTATAATTTTTTATTATTTTTAAATAAATTAA | *tkv allele switch* *cassette*  *tkv* 3’UTR-*loxP*-AscI |
| 23 | GGTGGCAGTTaCTTCAGCGCAG | C40Y mutation |
| 24 | GGGTCTGGTCTCGCAGGT | C40Y mutation |
| 25 | GAACATTGTCcGcTGCGACAAGGAGG | C90R mutation |
| 26 | TTGCCATGCAGGTGGGGT | C90R mutation |
| 27 | GGAGGACTTCcGgAACCGTGACC | C97R mutation |
| 28 | TTGTCGCAGCAGACAATG | C97R mutation |
| 29 | CATTGCCAAGgAtATTCAGATGGTG | Q199D mutation |
| 30 | GTTCTTTGCACCAGCAATG | Q199D mutation |
| 31 | GATGACCCGTtGCTGCTACACAC | R409C mutation |
| 32 | TCCCACAGAACGAGACCC | R409C mutation |
| 33 | GTTCGAGGACAcGCACGCTGTTG | M442T mutation |
| 34 | GTGGGATCCGAGGGCACC | M442T mutation |

**Supplementary File 1. Primers used in this study**

EcoRI and AscI sites are underlined in 17 and 22, respectively. Lowercase characters indicate point mutations.
